# Refractory Asthma Treatment Is Complicated by Tracheobronchomalacia: Case Reports and Review of the Literature

**DOI:** 10.1155/2013/735058

**Published:** 2013-05-13

**Authors:** Sawad Boonpiyathad, Atik Sangasapaviliya

**Affiliations:** Division of Allergy and Clinical Immunology, Department of Medicine, Phramongkutklao Hospital, Bangkok 10400, Thailand

## Abstract

Tracheobronchomalacia (TBM) is defined as the condition where the airway lumen narrows more than 50 percent. The acquired TBM usually occurs in adults; however, the prevalence of TBM in asthma is unknown. We report two cases of severe asthma in elderly patients that could not be controlled with higher medication use. Case 1 was a 70-year-old woman with sever persistent asthma for 10 years, presented with uncontrolled symptoms for 4 months. A CT of the chest showed collapse of the trachea at the posterior wall. Case 2 involved a 72-year-old woman with partly controlled asthma presenting with uncontrolled symptoms for 3 months. A CT of the chest showed normal distal tracheal anteroposterior diameter. However, bronchoscopy showed bronchomalacia at the right and left bronchus of the lower lungs. Patients who have severe asthma, despite adequate treatment with medication, should be further investigated to exclude other diseases that have clinical features similar to asthma such as tracheobronchomalacia, particularly in the elderly.

## 1. Introduction

Treatment-resistant severe asthma or refractory asthma is defined by uncontrolled asthma despite the highest level of recommended treatment. Only about 5% of total asthmatic patients have refractory asthma [1]. Adults with refractory asthma are more likely to have frequent severe asthma exacerbation, require treatment with oral corticosteroids, and visit the emergency department or be admitted to the hospital than those whose asthma is well controlled [2]. Other diseases have similar clinical symptoms such as chronic obstructive pulmonary disease (COPD) and severe asthma such as vocal cord dysfunction (VCD) and tracheobronchomalacia (TBM). 

TBM is a disease of the central airways characterized by a weakness of the tracheal and bronchial walls caused by the softening of supporting cartilage. As a result, the airway loses its stiffness and the walls move closer together, especially during expiration causing a reduction of at least 50% in the transverse area of the tracheal lumen [3]. The prevalence of TBM in adults is unknown. However, it appears to be more common among middle-aged and elderly men [4]. Patients with TBM may present symptoms of dyspnea, orthopnea, chronic cough, hemoptysis, wheezing, stridor, and hypercapnic respiratory failure [3, 5]. Bronchoscopic visualization of dynamic tracheal or bronchial collapse remains the gold standard for diagnosis [6] and dynamic expiratory computer tomography (CT) scanning is highly sensitive for detecting TBM [7]. We report 2 cases having TBM as a cause of refractory treatment asthma. 

## 2. Case Report

*Case  1.* The first case involved a 70-year-old nonsmoking woman with a history of severe persistent asthma for 10 years including diabetes mellitus, hypertension, dyslipidemia, and ischemic heart disease. In 2011, she was referred to our allergy clinic with severe asthma. In the past 4 months, her asthma symptoms could not be controlled and asthmatic attacks always happened at night and worsened while she was sleeping. She was treated with fluticasone/salmeterol accuhaler (250/50 mcg) 2 puffs, twice daily; montelukast (10 mg); sustained release theophylline (200 mg) 1 tablet, twice daily; procaterol (25 mcg), 1 tablet, twice daily; salbutamol evohaler, 2-3 times daily for dyspnea. On physical examination, she was obese and presented expiratory wheezing and stridor in the supine position. 

**Figure 1:**
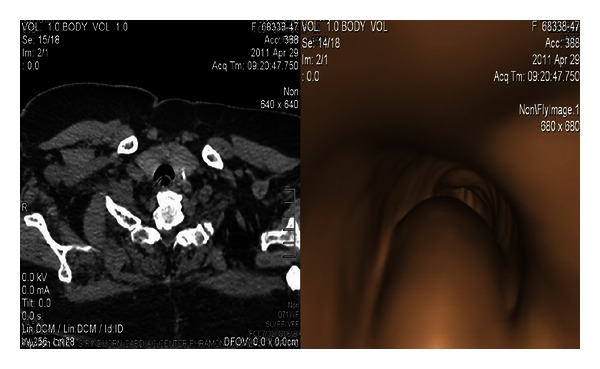
Chest dynamic multislice CT scan and CT virtual bronchoscopy showing collapse of trachea at posterior wall.

Chest radiography showed mild cardiomegaly, and parasinus radiography did not show sinus infection. Pulmonary function test (PFT) showed moderate restrictive lung disease and forced expiratory volume in the first second (FEV_1_) 59% predicted. To rule out cardiac problem, an echocardiogram was performed showing good left ventricular ejection fraction (LVEF) of 70% and marked concentric left ventricular hypertrophy. Additionally, chest dynamic multislice CT and CT virtual bronchoscopy were performed. Trachea showed collapse at the posterior wall (Figure 1), with a crescent type without any intraluminal or extraluminal mass. Tracheomalacia was diagnosed and she was treated with continuous positive airway pressure (CPAP) 5 cm H_2_O. Her asthma symptoms had improved without nocturnal dyspnea. Three months later, she could use only the fluticasone/salmeterol accuhaler for controlling her asthma symptoms.

*Case  2.* In 2010, a 72-year-old nonsmoking woman with a history of hypertension and severe persistent asthma for 15 years was referred to our allergy clinic for further treatment with omalizumab (anti-IgE). Skin prick test showed positive results against house dust and cat. Total IgE was 523 U/mL (35–70) after treatment with omalizumab 300 mg taken every two weeks for four months. Her asthma symptoms remained partly controlled (asthma control test (ACT) score 20 and peak expiratory flow rate (PEFR) 180 L/min). She was treated with fluticasone/salmeterol accuhaler (250/50 mcg) 2 puffs twice daily; montelukast (10 mg); sustained release using theophylline (200 mg); tiotropium (18 mcg) once daily; salbutamal evohaler. In the first 3 months of 2012, her asthma symptoms were uncontrolled (ACT score 7 and PEFR 100 L/min). She developed acute asthma exacerbation and was treated with oral prednisolone (20–30 mg) and increased fluticasone/salmeterol accuhaler (500/50 mcg) 2 puffs twice daily and showed no improvement. On physical examination, she was observed to be obese and presented tachypnea and expiratory wheezing of both lungs. 

**Figure 2:**
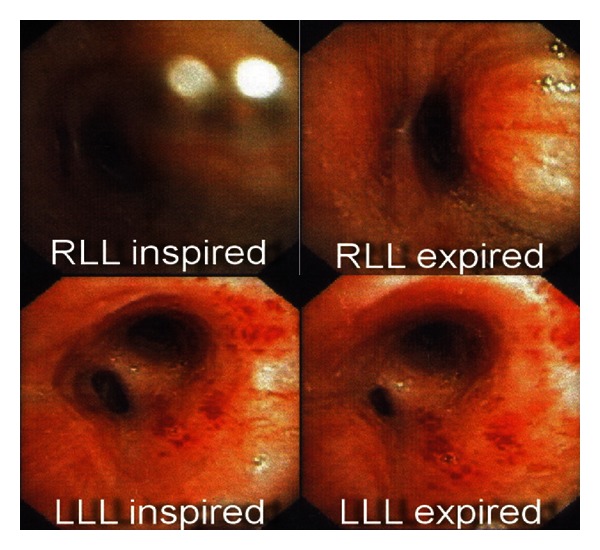
Bronchoscopy images during inspiration and expiration.

 Chest radiography showed mild cardiomegaly. PFT showed FEV_1_ 35% predicted and no response to the bronchodilator. Echocardiogram showed LVEF 53% with no abnormal wall movement. We consulted an otolaryngologist to evaluate vocal cord movement and other upper respiratory tract obstruction. No vocal cord dysfunction or other obstructions were observed. Chest CT was normal while bronchoscopy showed that the right and left bronchus of lower lungs were narrow. Bronchomalacia was diagnosed as shown in Figure 2. She denied with invasive treatment. She was treated with breath training exercise and given CPAP, 5 cm H_2_O at bedtime.

## 3. Discussion

In making the diagnosis of refractory asthma, other diseases are important to consider and to exclude other diseases in the differential diagnosis of wheeze, dyspnea, cough, and eosinophilia. Specifically, patients should be evaluated for other diseases such as COPD, allergic bronchopulmonary aspergillosis (ABPA), cystic fibrosis, VCD, Churg-Strauss syndrome (CSS), and TBM. Furthermore, many comorbid conditions may occur with asthma especially gastro-esophageal reflux disease (GERD), rhinitis/sinusitis, psychological disturbances, chronic infections, obstructive sleep apnea (OSA), and obesity [8]. Interaction of these comorbidities with asthma may be complex, so they may complicate the course of asthma or may act as confounding factors in the diagnosis or assessment of asthma control.

 The primary form of TBM is congenital, and the natural history is usually one of gradual improvement because the airway lumen increases in diameter and cartilage becomes more rigid as the child ages. Acquired forms of TBM are disorders of middle-aged and elderly people [9]. Acquired TBM is caused by the degeneration of normal cartilaginous support from a variety of causes [10]. The most common posttraumatic causes of acquired TBM include tracheostomy, intubation with endotracheal tube, and chest trauma [6]. Other causes of acquired TBM are COPD, chronic inflammation (relapsing polychondritis), chronic external compression of trachea (malignancy, benign tumors, cysts, abscesses, and aortic aneurysm), and vascular ring [11]. Patients with TBM presented with wheezing, 51%, and wheezing with dyspnea, 17%. They were usually resistant to treatment with corticosteroid and bronchodilator [12] whose symptoms were often confused with refractory asthma. Of 80 patients with suspected or proven TBM, 40% were found to have COPD and 24% asthma [13]. Recently, Kandaswamy et al. have documented a high prevalence of TBM in patients admitted to ICU with recurrent hypercapnic respiratory failure [5]. Adult asthmatic patients who had comorbidity with TBM remain unknown; however, a recent case controlled study in ICU has shown that severe GERD and obesity are associated with TBM [5]. Few cases have reported that TBM was the cause of severe asthma [14, 15]. The causes of TBM in our patients remain unknown but maybe from a degenerative change of the cartilage. In addition, they had similar risk factors, that is, being a woman, old age, obesity, long-time use of inhaled corticosteroid, and prolonged duration of asthma. Moreover, TBM is usually determined under radiographic detection because traditional imaging studies performed at end inspiration do not allow for an assessment of airway collapsibility. Bronchoscopy has generally been considered as the gold standard for diagnosis; however, it is an invasive method. Advances in technology such as dynamic multislice helical CT, has improved the ability to assess TBM noninvasively. It provides an intraluminal perspective and improves correlation with bronchoscopy [16]. 

 Many adult patients with TMB do not require therapy because the finding may be incidental, and these diseases may not cause symptoms. The first line of treatment is to control the symptoms of the concomitant underlying diseases. These include stopping cigarette smoking, effectively treating respiratory infections, rehabilitation, breathing, and relaxation techniques, nonpharmacological measures for gastroesophageal reflux, treatment of the disorders in the upper respiratory tract, and identification and treatment of OSA [6]. Noninvasive ventilation with CPAP can be used to maintain airway patency, facilitate secretion drainage, and improve expiratory flow. CPAP acts as a pneumatic stent, decreases pulmonary resistance, and improves expiratory airflow obstruction [17]. Few studies have shown that spirometry values improved during the acute administration of nasal CPAP [18]. These patients had improved sputum production and atelectasis, improved exercise tolerance, and reduced need for long-term medical care [18]. Airway stents are often able to restore and maintain airway patency in patients with any form of central airway obstruction. PFT improves significantly after stenting of various causes of central airway obstruction, including malacia [19]. Open surgery such as tracheostomy, airway splinting, and treachal resection should be employed in the selected case for functionally disabled TBM that fail other therapies [4].

In conclusion, asthma refractory to treatment should alert clinicians to the differential diagnosis of other reasons for airway obstruction. TBM is underdiagnosed because its symptoms are similar to that of severe asthma. Chest CT scan and bronchoscopy are employed to investigate the cause of severe asthma especially in the elderly. Treatment comorbid conditions of severe asthma could improve asthma symptoms leading to a better quality of life.

## Abbreviations

TBM: Tracheobronchomalacia
CT: Computer tomography
PFT: Pulmonary function test
FEV_1_: Expiratory volume in the first second
LVEF: Left ventricular ejection fraction
PEFR: Peak expiratory flow rate
ACT: Asthma control test.
